# Accumulation and aberrant composition of cholesteryl esters in Scrapie-infected N2a cells and C57BL/6 mouse brains

**DOI:** 10.1186/1476-511X-10-132

**Published:** 2011-08-04

**Authors:** Sarah Vascellari, Sebastiano Banni, Claudia Vacca, Vito Vetrugno, Franco Cardone, Michele A Di Bari, Paolo La Colla, Alessandra Pani

**Affiliations:** 1 Department of Biomedical Science and Technology, University of Cagliari, 09042-Monserrato, Italy; 2 Department of Experimental Biology, University of Cagliari, 09042-Monserrato, Italy; 3 Department of Cell Biology and Neurosciences, Istituto Superiore di Sanità, Viale Regina Elena 299, 00161-Rome, Italy; 4 Department of Animal Health, Istituto Superiore di Sanità, Viale Regina Elena 299, 00161-Rome, Italy

**Keywords:** Prions, Cholesterol, Cholesteryl esters, Fatty acids, Statins

## Abstract

**Objective:**

Cholesterol changes have been described in prion-cell models and in experimental rodent scrapie; yet, the pattern of this association is still controversial.

**Methods:**

To shed light on the matter, we analysed and compared cholesterol variations in ScN2a cells and in brains of Scrapie-infected C57Bl/6 mice, using two different methods: a fluorimetric-enzymatic cholesterol assay, and high performance liquid chromatography-mass spectroscopy (HPLC-MS).

**Results:**

Compared to uninfected controls, similar cholesterol metabolism anomalies were observed in infected cells and brains by both methods; however, only HPLC-MS revealed statistically significant cholesterol variations, particularly in the cholesteryl esters (CE) fraction. HPLC-MS analyses also revealed different fatty acid composition of the CE fraction in cells and brains. In N2a cells, their profile reflected that of serum, while in normal brains cholesteryl-linoleate only was found at detectable levels. Following prion infection, most CE species were increased in the CE pool of ScN2a cells, whereas a conspicuous amount of cholesteryl-arachidonate only was found to contribute to the cerebral increase of CE. Of interest, oral pravastatin administration to Scrapie-infected mice, was associated with a significant reduction of cerebral free cholesterol (FC) along with a concomitant further increase of the CE pool, which included increased amounts of both cholesteryl-linoleate and cholesteryl-arachidonate.

**Conclusion:**

Although mechanistic studies are needed to establish the pathophysiological relevance of changes in cerebral CE concentrations, to the best of our knowledge this is the first report to provide evidence of increased cholesterol esterification in brains of prion-infected mice, untreated and treated with pravastatin.

## Introduction

It is now accepted that modifications of cholesterol concentrations are linked to prion infection/replication [1-4]; yet, no general agreement on the precise prion-associated cholesterol concentration changes, as well as on the relevance of cholesterol-lowering drugs in the control of prion diseases, has been reached. *In vitro*, some studies produced evidence that cholesterol depletion abolishes prion protein (PrP)-raft association, promotes PrP accumulation, and increases substantially its misfolding into the pathologic scrapie-prion protein isoform (PrPSc) [5,6]. On the other hand, although the majority of *in vivo *studies failed to link statins' prophylactic effect to a reduction of the bulk of cerebral cholesterol [7-12], the lowering of cholesterol with statins has been reported to inhibit PrPSc generation in cell-based prion models [13,14]. More than just changes in cholesterol contents, prion infection seems to be accompanied by a general derangement of cholesterol homeostatic mechanisms [15,16], possibly triggered by prion itself [4]. In our previous studies, increased levels of free cholesterol (FC) and of the cholesterol fraction esterified with free fatty acids (CE) were the main modifications observed. In prion-infected ScN2a cells, a number of drugs that indirectly targeted cholesterol esterification by affecting steps of cholesterol metabolism/trafficking other than biosynthesis, were associated with a reduction of the CE pool and selective anti-prion activity [17]. Furthermore, selected combinations of these cholesterol-modulating drugs produced strong synergistic anti-prion effects, apparently by restoring cholesterol homeostasis [18]. Somewhat contradictory to our findings, however, other research groups reported that the increased content of FC in prion-infected neuronal cell lines was associated with a reduced content of CE [19,20]. Since the understanding of the mechanism(s) that regulate the structural conversion of PrP into pathogenic isoform(s) remain a fundamental target also for the development of novel therapeutic approaches, it is crucial to elucidate alterations in cholesterol metabolism associated with prion infection. In addition to discrepancies due to the different prion models employed to study such a complex relationship, the use of various methods for cholesterol measurements may also have contributed to some of the present conflicting findings. In order to clarify these inconsistent results, qualitative and quantitative cholesterol variations following Scrapie infection were analysed and compared in brains of C57BL/6 mice, untreated and treated with pravastatin (PRV), as well as in N2a cell lines. Two methods were used: fluorimetric-enzymatic Amplex Red cholesterol assay, and high performance liquid chromatography with mass spectrometry (HPLC-MS).

## Materials and methods

### Chemicals

Chloroform, methanol, N-heptane, di-isopropyl ether, formic acid, acetonitrile, and iodine bisublimate were purchased from Carlo Erba (Italy). Pravastatin (PRV) sodium salt was kindly provided by Bristol-Myers Squibb.

### Cell lines

The mouse neuroblastoma N2a cell line and a sub-line persistently infected with the mouse-adapted 22L-strain of scrapie (ScN2a cells), were a generous gift of Byron Caughey, Rocky Mountain Laboratories, NIAID-NIH, Hamilton MT, USA. Cell lines were grown and maintained at 37°C and 5% CO_2 _in OptiMEM supplemented with 10% bovine serum (Gibco-Invitrogen, Italy), 2 mM L-glutamine, 50 U/ml penicillin G sodium, and 50 μg/ml streptomycin sulphate (Gibco-Invitrogen, Italy), and splitted every 3 to 4 days. Cell lines were replaced every three months with freshly towed cells from liquid nitrogen. All experiments were carried out in exponentially growing cells harvested as monolayers reached sub-confluence (80-90%).

### Mice

C57BL/6 mice were scrapie-infected and PRV-treated as previously reported [21]. Briefly, one-month-old female C57BL/6 mice (Charles River) weighing 18-20 g, were inoculated intracerebrally (i.c.) in the left hemisphere with 1% (w/v) brain homogenate prepared from terminally ill, strain 139A scrapie-infected mice, and assigned randomly to the untreated (n = 4) or the PRV-treated (n = 4) group. Five mice were mock-inoculated (control group, n = 5). PRV (mouse oral LD_50_, 8939 mg kg^-1^) was administered in the drinking water at a dose of 200 mg (kg body weight)^-1 ^day^-1 ^from the time of scrapie inoculation. Mice were fed ad libitum with standard chow diet. Water consumption was monitored twice weekly and drug concentration was adjusted as required. Control and untreated animals received water without PRV. Terminally ill mice were killed by cervical dislocation under CO_2 _narcosis. Brains were collected, divided into the two hemispheres, and kept at -80°C. Hemi-brains were thawed, weighed, and homogenized in nine volumes (10% w/v) of phosphate-buffered saline (PBS, Invitrogen) and 0,1% Triton X-100 by sonication pulses (Vibra Cell, Sonics & Materials Inc., Newtown, CT) on ice, then boiled for 15 min to inactivate endogenous cellular cholesterol esterase. The resulting 10% homogenate was stored at - 80°C until lipid extraction. Mice, individually identified by a passive integrated transponder, were treated according to Legislative Decree 116/92 guidelines. Animal welfare was routinely checked by veterinarians from the Service for Biotechnology and Animal Welfare of the Istituto Superiore di Sanità. The research protocol has been approved by the Service for Biotechnology and Animal Welfare of the Istituto Superiore di Sanità and authorized by the Italian Ministry of Health, according to Legislative Decree 116/92 (Decreto Legislativo, 1992), which has implemented in Italy the European Directive 86/609/EEC (Council of the European Communities, 1986) on laboratory animal protection.

### Lipid Extraction from cell cultures

N2a and ScN2a cells were seeded at density of 1 × 10^5^cells/ml in T-75 flasks. After four day incubation at 37°C in a humidified 5% CO2 atmosphere, sub-confluent cultures were trypsinized and washed twice in sterile PBS to eliminate residual growth medium. Cells were resuspended in PBS, counted in a haemocytometer, and viable cells divided into three aliquots. One aliquot of 1 × 10^6 ^cells was used for protein determination by the bicinchoninic acid protein assay (Sigma). The other two aliquots were subjected to lipid extraction by the Folch method [22]. Total lipid extracts were dried in an evaporator and re-suspended in methanol for HPLC-MS analysis, or in Reaction buffer for Amplex Red cholesterol assay. HPLC-MS analysis were performed on extracts from a total of 30-40 × 10^6 ^cells. Amplex Red determinations were performed on extracts from a total of 4-5 × 10^6 ^cells.

### Lipid Extraction from brain homogenates

Lipids were extracted from brain homogenates by the Folch method [22]. In brief, brain homogenates were mixed with chloroform-methanol (2:1 v/v). Vitamin E was added as antioxidants to prevent lipid degradation during analysis. After 1 hour incubation in the dark, water was added (Clor/MeOH/H2O, 2:1:1 v/v/v) and samples were incubated for an additional hour in the dark. Samples were centrifuged at 1500 rpm for 1 hour at room temperature, then the lipid-containing organic phase was recovered. Total lipid extracts were dried in an evaporator and re-suspended in methanol for HPLC/MS analysis.

### HPLC-MS cholesterol analysis

Separation of free cholesterol and cholesteryl esters was carried out as described [23] with an Agilent 1100 HPLC system (Agilent, Palo Alto, CA) equipped with a diode array and mass spectrometer detectors in line. A C-18 Inertsil 5 ODS-2 Chrompack column (Chrompack International BV, Middleburg, the Netherlands), 5 μm particle size, 150. 4.6 mm was used. FC and CEs were detected at 200 nm. Spectra (195-315 nm) of the elute were obtained every 1.28 s, and were electronically stored. These spectra were taken to confirm the identification of the HPLC peaks. MS analysis was performed using a single quadrupole mass spectrometer (Agilent, Palo Alto, CA) equipped with an atmospheric Pressure chemical ionization source. Each sample was triplicated in the assays and at least three independent experiments were performed.

### Amplex Red cholesterol assay

Cholesterol content in cell and brain extracts was evaluated by the Amplex Red Cholesterol Assay kit (Invitrogen) according to the manufacturer's instructions. Briefly, mouse brain and cell extracts were diluted 1:10 with 1× cholesterol reaction buffer (0.1 M potassium phosphate, pH 7.4, 0.05 M NaCl, 5 mM cholic acid, 0.1% Triton X-100). Fifty microliters of 150 μM Amplex Red reagent (1 U/ml horseradish peroxidase, 1 U/ml cholesterol oxidase, and 1 U/ml cholesterol esterase) were added to 50 μL sample in 96 well plates. After 60-min incubation at 37°C in the dark, sample fluorescence was measured using a microplate reader (Victor 3V 1420 Multilabel Counter, Perkin Elmer) at 530/25 nm excitation, and 590/35 nm emission wavelengths. The total cholesterol (TC) content was determined by measuring the cholesterol concentration following digestion with cholesterol esterase. To measure FC, cholesterol esterase was omitted from the assay. Each sample was triplicated in the assay and at least three independent experiments were performed. Values obtained from a cholesterol standard curve were normalized to 10^6^cells (N2a and ScN2a cells) or to g of tissue (brains).

### Statistical analysis

All statistical comparisons were calculated using a two-way, unpaired Student's t-test. Statistical comparisons among groups were also made by using a one-way ANOVA, and where appropriate a post hoc Bonferroni test. Statistical significance was assigned to *p *< 0.05. All values are expressed as mean values ± standard deviation (SD).

## Results

### Amplex Red vs. HPLC-MS cholesterol analyses in N2a cell and C57Bl/6 mouse prion-models

Cholesterol measurements by Amplex Red showed a significant increase in the FC content (*p *= 0.015) in ScN2a cells compared to uninfected N2a cultures. Despite a tendency to increase, total cholesterol (TC) and CE levels did not significantly differ between infected and uninfected cells (*p *= 0.364 and *p *= 0.664, respectively) when determined by this method (table 1). By contrast, HPLC-MS analysis showed that TC, FC and CE levels were all significantly increased (*p *≤ 0.006) in the ScN2a cell extracts (table 1).

**Table 1:** Amplex Red vs. HPLC-MS cholesterol composition in N2a and ScN2a cells.

Method	Cell lines	nmol/10^6 ^cells			CE/TC %
		TC	FC	CE	
**Amplex Red**	**N2a**	80.11 ± 4.18	77.52 ± 0.38	2.59 ± 1.92	3.23
	**ScN2a**	83.60 ± 4.18	79.80 ± 0.88*	3.81 ± 4.08	4.55
		*p = 0.364*	***p = 0.015***	*p = 0.664*	
**HPLC-MS**	**N2a**	135.36 ± 13.59	98.38 ± 9.75	36.98 ± 4.01	27.0
	**ScN2a**	200.37 ± 8.27*	146.54 ± 4.76*	53.83 ± 3.60*	26.8
		***p = 0.002***	***p = 0.002***	***p = 0.006***	

Total (TC), free (FC) and esterified (CE) cholesterol in uninfected (N2a) and persistently prion-infected (ScN2a) cell extracts. Values represent the mean ± SD of triplicate determinations and are representative of at least three independent trials. *Statistical significance by unpaired Student's t-test.

Given that lipid variation in N2a cell cultures may be influenced by the serum in the culture medium, TC, FC, and CE levels were also determined by the two methods in brains of uninfected and terminally ill scrapie-infected C57Bl/6 mice, untreated or treated with PRV (200 mg/kg bw/day). Again, TC, FC and CE levels determined by Amplex Red did not significantly differ between normal and scrapie-infected brains (*p *> 0.3) (table 2). Instead, when mouse brain extracts were subjected to HPLC-MS analysis, the increase of cerebral CE in the scrapie-affected group were highly significant (*p *= 0.008). In addition, consistent with CE/TC percentages commonly found in tumour cell lines and normal brains [24], direct measurements by HPLC-MS revealed approximately 27% CE in uninfected N2a cells, and approximately 10% CE in normal mouse brains (table 1 and 2). In contrast, Amplex Red measurements underestimated percentages of CE/TC, both in cells (< 5%) and brains (< 1%). The HPLC-MS data also allowed us to show that, compared to normal brains, CE/CT percentages significantly increased (*p *= 0.029) in brains of the scrapie-affected mouse group (from 9.2% to 12.3%), while they did not vary in N2a and ScN2a cells (27.0% vs. 26.8%, respectively) (tables 1 and 2).

**Table 2:** Amplex Red vs. HPLC-MS cholesterol compositions in brains of uninfected and Scrapie-infected C57BL/6 mice.

Method	Mouse brains	μ mol/g tissue			CE/TC %
		TC	FC	CE	
**Amplex Red**	**Uninfected**	276.38 ± 11.78	275.24 ± 14.44	1.14 ± 1.24	0.41
	**Scrapie-infected**	283.48 ± 8.74	282.24 ± 9.88	1.24 ± 1.59	0.43
		*p = 0.351*	*p = 0.438*	*p = 0.918*	
**HPLC-MS**	**Uninfected**	124.35 ± 29.61	112.87 ± 17.37	11.48 ± 3.26	9.23
	**Scrapie-infected**	156.58 ± 25.46	137.24 ± 16.70	19.33 ± 3.20*	12.34
		*p = 0.129*	*p = 0.071*	***p = 0.008***	

Total (TC), free (FC) and esterified (CE) cholesterol in brain extracts of uninfected (n = 5) and 139A-scrapie infected (n = 4) mice. Values represent the mean ± SD of triplicate determinations and are representative of at least three independent trials. *Statistical significance by unpaired Student's t-test.

According to our previous study [21], oral long-term and high-dose PRV-treatment of scrapie-infected mice confirmed a moderate prophylactic effect (data not shown). As would be expected for an inhibitor of cholesterol biosynthesis, a consistent reduction of FC was observed in brains of PRV-treated vs. untreated Scrapie-infected mice (table 3). Once again, it must be emphasized that statistically significant FC differences between the drug-treated and the untreated group of infected mice were revealed by HPLC-MS analyses (*p *= 0.026), but not by Amplex Red (*p *= 0.432). Unexpectedly, in the PRV-treated scrapie-affected group, CE appeared to be further increased (table 3). Statistical analysis performed by using one-way ANOVA test showed significant differences in FC and CE (*p *= 0.047 for FC, and *p *= 0.001 for CE) among the three experimental groups, i.e., uninfected, scrapie-infected, and scrapie-infected PRV-treated. Of note, the opposite change in the content of FC, reduced, and CE, increased, by this drug may explain why statistically significant variations in cholesterol levels between untreated and PRV-treated scrapie-infected mice have not previously been revealed [10].

**Table 3:** Amplex Red vs. HPLC-MS cholesterol compositions in brains of untreated and pravastatin-treated Scrapie-infected C57BL/6 mice.

Method	Scrapie-infected mouse brains	μ mol/g tissue			CE/TC %
		TC	FC	CE	
**Amplex Red**	**Untreated**	283.48 ± 8.74	282.24 ± 9.88	1.24 ± 1.59	0.43
	**PRV-treated**	279.12 ± 6.84	276.68 ± 8.74	2.44 ± 1.27	0.87
		*p = 0.462*	*p = 0.432*	*p = 0.283*	
**HPLC-MS**	**Untreated**	156.58 ± 25.46	137.24 ± 16.70	19.33 ± 3.20	12.34
	**PRV-treated**	134.17 ± 27.74	106.03 ± 13.46*	28.14 ± 6.62*	20.97
		*p = 0.247*	***p = 0.026***	***p = 0.05***	

Total (TC), free (FC) and esterified (CE) cholesterol in brain extracts of 139A-scrapie infected (n = 4), and of 139A-scrapie-infected C57Bl/6 mice treated with pravastatin (PRV, 200 mg/kg b.w.). Values represent the mean ± SD of triplicate determinations and are representative of at least three independent. *Statistical significance by unpaired Student's t-test.

### Fatty acid composition of CE in uninfected and prion-infected N2a cell and C57BL/6 brain extracts

**Figure 1:**
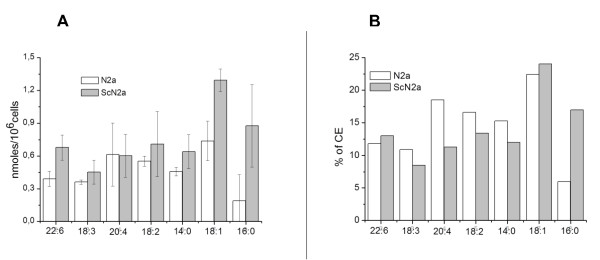
**HPLC-MS composition of fatty acids esterified to cholesterol in N2a and ScN2a cells**. A) concentration of oleic (C18:1), arachidonic (C20:4), linoleic (C18:2), miristic (C14:0), docosahexaenoic (C22:6), α-linolenic (C18:3), palmithic (C16:0) acid expressed in nanomoles/10^6 ^N2a (uninfected) and ScN2a (prion-infected) cells. Values represent the mean ± SD of triplicate determinations and are representative of at least three independent trials; B) each fatty acid as percentage of total cholesteryl esters (CE).

The HPLC-MS lipid analyses allowed us to identify and measure various CEs based on the fatty acid (FA) esterified to cholesterol in our prion-models (Figure 1a and 1b). In uninfected N2a cells, the FA esterified to cholesterol, from most to least abundant, were: oleic (C18:1) > arachidonic (C20:4) > linoleic (C18:2) > miristic (C14:0) > docosahexaenoic (C22:6) > α-linolenic (C18:3) > palmithic (C16:0) (Figure 1a). With the exception of CE 20:4, all CE species quantitatively increased in ScN2a cells; with C16:0, C18:1, and C22:6 being the most elevated (Figure 1a). With respect to total CE, however, higher proportions of C16:0, lower proportions of C20:4, C18:3, C18:2, and C14:0, and slight or no variations in C22:6 and C18:1 were observed in ScN2a compared to N2a cells (Figure 1b).

**Figure 2:**
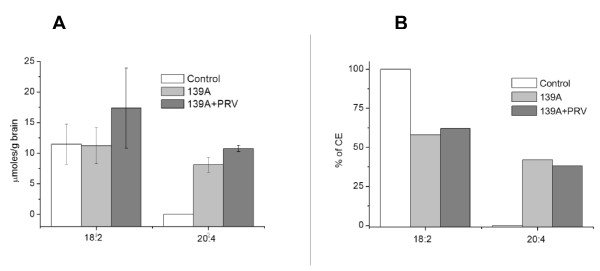
**HPLC-MS composition of fatty acids esterified to cholesterol in brain extracts of uninfected, scrapie-infected, and scrapie-infected PRV-treated C57BL/6 mice**. A) concentration of linoleic (C18:2) and arachidonic (C20:4) acid expressed in micromoles/g of brain tissue. Values represent the mean ± SD of triplicate determinations and are representative of at least three independent trials; B) each fatty acid as percentage of total cholesteryl esters (CE). Variation of C20:4 among groups, i.e. uninfected, Scrapie-infected, and PRV-treated Scrapie-infected, is statistically significant by ANOVA (*p *= 0.001).

Analyses of mouse brains showed an entirely different CE composition (Figure 2a and 2b). The only CE identified in uninfected brains was CE 18:2. In scrapie-affected brains, despite a significant increase in the CE fraction, the level of CE 18:2 did not vary. Furthermore, a consistent amount of CE 20:4 was detected. Interestingly, an increase of both CE 18:2 and CE 20:4 appeared to characterize the CE pool of PRV-treated scrapie-affected brain extracts. The ANOVA test showed a highly significant variation of C20:4 (*p *= 0.001) among the three experimental groups, i.e. uninfected, scrapie-infected, and PRV-treated scrapie-infected brains.

## Discussion

In the current study, qualitative and quantitative lipid analyses by HPLC-MS revealed multiple anomalies associated with prion infection both in ScN2a cell cultures and scrapie-affected C57Bl/6 mouse brains, mainly consisting in abnormal accumulation of CE. These results are in agreement with our previous findings [17,18], as well as with those of a recent systems biology study showing that the *SOAT1 *gene, encoding for the enzyme (i.e. ACAT1) responsible for cholesterol esterification, is the first of all cholesterol-related genes to be up-regulated in the brain of scrapie-infected mice [25]. To date, few studies have tried to dissect variations in intracellular FC versus CE pools [reviewed in 16], and no univocal hypothesis yet exists on the increase of cytoplasmic CE in prion-infected cells. As a matter of fact, some authors using the fluorimetric-enzymatic cholesterol assay Amplex Red reported that prion-infected neuronal cell lines are characterized by a reduced content of CE [19,20]. In our hands, however, even the Amplex Red cholesterol measurements showed a higher, though not statistically significant, CE content in ScN2a vs. N2a cells. Mixed results may be accounted for by the fact that Amplex Red directly measures only the TC and FC, while the CE fraction is indirectly calculated by subtraction of FC from TC. Given that the percentage of CE in TC is known to be always markedly low (between 10% and 30% depending on the type of tissue) [24], the experimental error using the Amplex Red method can, in fact, become substantial [26]. Actually, we found that CE levels calculated by Amplex Red were up to 90% lower than those obtained by HPLC-MS, both in N2a cells and mouse brains. Therefore, enzymatic assays seem suitable for the rapid analysis of samples high in cholesterol content (i.e. plasma), but less appropriate to ascertain cholesterol concentration changes in other types of samples, especially in those cells and tissues where CE levels are particularly low. Also, it has to be pointed out that, with respect to TC and FC content, even though Amplex Red showed higher levels in all prion-infected samples compared to uninfected controls, only HPLC-MS data showed statistically significant differences. Given that HPLC-MS identifies and quantifies the different CE species, we were also able to demonstrate that an entirely different composition of FA families characterized the CE fraction of cultured neuronal cells and mouse brains. Consistent with the lipid composition of bovine serum [27,28], C18:1, C20:4, C18:2, C14:0, C22:6, C18:3, and C16:0 FA families were detected in the CE fraction of cultured N2a cells. By contrast, in normal brains only cholesteryl-linoleate was detected, probably because C18:2 is by far the most abundant free FA in the standard mouse chow diet [29]. Following Scrapie infection, besides CE 18:2 (52%), a conspicuous amount of CE 20:4 (48%) was found to compose cerebral CEs. Since a major pathway for the production of arachidonic acid (C20:4) is the deacylation of phospholipids in the sn-2 position by phospholipases A_2 _(PLA_2_) [30], it is possible to speculate that the C20:4 found as an integral component of CE in Scrapie-infected brains was released from membranes' phospholipids by the action of PLA_2_. Actually, prions have recently been reported to trigger abnormal activation of the cytoplasmic-PLA_2 _(c-PLA_2_) [20,31]. The increased cholesterol esterification may then represent an attempt by the infected cell to reduce concentrations of a potent inflammatory factors' precursor. Another interesting result of this study was that high-dose and long-term oral PRV-treatment of Scrapie-infected C57BL/6 mice, able to delay disease progression and to prolong survival times [21], was associated with reduction of cerebral FC along with a concomitant further increase of the CE fraction. Intriguingly, in the PRV-treated group, both CE 18:2 (63%) and CE 20:4 (37%) appeared to contribute to the further increase of CE content in brains. Mechanistic *in vivo *studies are needed to establish the pathophysiological relevance and the dynamic of these changes in lipid metabolism, especially to define their consequences in terms of production of inflammatory mediators and alteration of membrane lipid domains [31,32]. Nonetheless, to the best of our knowledge, this may be the first report that provides evidence of an accumulation of CE in brains of Scrapie-infected mice. Moreover, we show an effect of pravastatin on the levels of cerebral CE that has not been previously reported: a drug-induced amplification of the esterification of cholesterol in the brain. These findings could be even more relevant considering that all, or nearly all, cerebral cholesterol derives from *de novo *synthesis, mostly present in the un-esterified form [33-35]. In addition, the differences found in the FA composition of CE in neuronal cells and brains, as well as their different changes after Scrapie infection, raise the question of whether cultured cells should represent the model of choice for the comprehension of the mechanistic link between lipid alterations and the pathogenesis of prion diseases.

## Conclusions

The characterization of cholesterol changes during prion infection is crucial to the understanding of the mechanisms that govern prion generation, and developing of effective therapies. To date, no univocal hypothesis exists on either modifications of free vs. esterified cholesterol, or the statins' cholesterol-lowering effect in rodent Scrapie. Conflicting and inconclusive data may be due to different prion models, and the use of dissimilar methods for cholesterol measurements. Our study advances present knowledge as it: i) reveals the critical importance of the cholesterol measuring method; ii) demonstrates that the cholesterol esterification pathway is enhanced following prion infection; iii) illustrates the previously unknown promoting effect of pravastatin on esterification of cerebral cholesterol; iv) shows that the increased CE fraction in infected brains is due to esterification of free cholesterol to arachidonic acid, major precursor of inflammatory mediators; and, v) warns against the use of cultured cells to unveil the link between lipids and prions.

## Competing interests

The authors declare that they have no competing interests.

## Authors' contributions

SV, PLC, and AP designated the study. SV performed cell and brain extracts, immunodetection of cellular and PK-resistant prion protein, all enzymatic cholesterol determinations, statistical analysis of data, and drafted the manuscript. SB and CV performed all HPLC-MS analyses. VV, FC, and MAD performed the *in vivo *experiments and collected biological samples. AP wrote the final manuscript. All authors read and approved the final manuscript.
